# Electrophysiology Alterations in Primary Visual Cortex Neurons of Retinal Degeneration (S334ter-line-3) Rats

**DOI:** 10.1038/srep26793

**Published:** 2016-05-26

**Authors:** Ke Chen, Yi Wang, Xiaohua Liang, Yihuai Zhang, Tsz Kin Ng, Leanne Lai Hang Chan

**Affiliations:** 1 Key Laboratory for Neuroinformation of Ministry of Education, School of Life Science and Technology, University of Electronic Science and Technology of China, Chengdu, China; 2 Department of Electronic Engineering, City University of Hong Kong, Hong Kong; 3 Department of Ophthalmology and Visual Sciences, The Chinese University of Hong Kong, Hong Kong; 4 Centre for Biosystems, Neuroscience, and Nanotechnology, City University of Hong Kong, Hong Kong

## Abstract

The dynamic nature of the brain is critical for the success of treatments aimed at restoring vision at the retinal level. The success of these treatments relies highly on the functionality of the surviving neurons along the entire visual pathway. Electrophysiological properties at the retina level have been investigated during the progression of retinal degeneration; however, little is known about the changes in electrophysiological properties that occur in the primary visual cortex (V1) during the course of retinal degeneration. By conducting extracellular recording, we examined the electrophysiological properties of V1 in S334ter-line-3 rats (a transgenic model of retinal degeneration developed to express a rhodopsin mutation similar to that found in human retinitis pigmentosa patients). We measured the orientation tuning, spatial and temporal frequency tunings and the receptive field (RF) size for 127 V1 neurons from 11 S334ter-3 rats and 10 Long-Evans (LE) rats. V1 neurons in the S334ter-3 rats showed weaker orientation selectivity, lower optimal spatial and temporal frequency values and a smaller receptive field size compared to the LE rats. These results suggest that the visual cognitive ability significantly changes during retinal degeneration.

Several major outer retinal diseases, such as retinitis pigmentosa (RP) and age-related macular degeneration, are caused by the degeneration of photoreceptors, which results in the loss of visual signals and major remodelling of the retinal circuitry^1^,^2^,^3^,^4^. Although potential treatments exist for outer retinal diseases, to date these diseases cannot be effectively cured^5^,^6^. The PDE6B gene encodes cGMP-phosphodiesterase in the rods, which is an essential protein in the phototransduction cascade; mutations in this gene usually cause RP in humans^7^,^8^. The S334ter-3 rat is a transgenic model of retinal degeneration (RD) developed to express a rhodopsin mutation similar to that found in human retinitis pigmentosa patients^9^. Therefore, the S334ter-3 rat is considered a suitable animal model to investigate the progression of degeneration in outer retinal diseases. Based on a previous study in the S334ter-3 model, the degeneration of photoreceptors begins in the central retina and progresses towards the periphery. Horizontal and rod bipolar cells show normal morphologies in the retinas on post-natal day 15 (P15). However, at P21 the horizontal and rod bipolar cells exhibit abnormal processes in the outer plexiform layer, whereas the outer nuclear layer is significantly thinner. A glial reaction occurs concomitantly. In contrast, modifications in cone-bipolar and amacrine cells are much slower and do not occur until P90 and P180, respectively. The density of the horizontal and rod-bipolar cells significantly drops after P60^10^.

Many previous studies have investigated the morphological and functional changes that occur in retinal cells in different degeneration models^11^,^12^,^13^,^14^,^15^,^16^,^17^,^18^. However, few studies have investigated the changes in the electrophysiological properties of the primary visual cortex during the course of RD. Investigations in LE rats discovered that the majority of neurons showed sharply tuned orientation selectivity with a bias for horizontal stimuli. Responses were elicited by spatial frequencies ranging from zero to 1.2 cycles per degree (cpd), and the optimal stimuli velocities varied from 10 to 250 degrees per second^19^. Orientation-specific interactions that occurred between the centre and the periphery of the receptive fields led to strong inhibition of centre stimulation when both stimuli had the same orientation^20^,^21^. Therefore, in this present study we measured functional changes in V1 using grating stimuli in the degenerated group (S334ter-3).

By conducting the extracellular recording, we measured the orientation tuning, spatial and temporal tunings, and RFs for each neuron from 11 S334ter-3 and 10 LE rats. Our results showed the V1 neurons in the degenerated group exhibited weaker orientation selectivity, lower optimal spatial and temporal frequency values, and smaller RFs compared to the control group.

## Results

In summary, we recorded 127 visually responsive cells from 11 S334ter-3 and 10 LE immature rats from 55–85 days of age. We recorded 5–10 neurons in one penetration. For single neurons, a battery of tests was performed to study the orientation tuning, spatial and temporal frequency, RF and surround suppression.

### Degeneration in the S334ter-3 retina and reconstruction of recording tracks in the primary visual cortex

**Figure 1:**
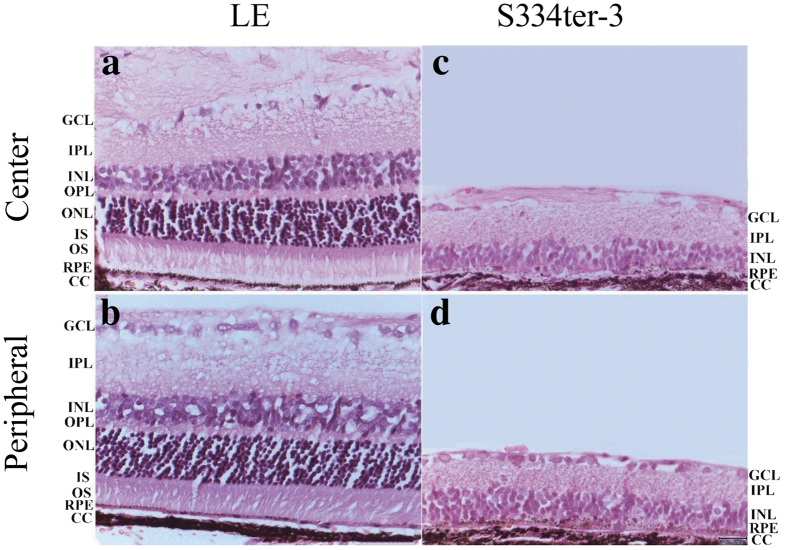
Light microscopy with H&E staining from a normal retina and a degenerated retina (P65). Nine laminar retinal layers are clearly visible in the centre (**a**) and peripheral (**b**) LE retinas. Retinal thickness is reduced in the S334ter-3 rat retinas with the absence of photoreceptor layers both in the central (**c**) and peripheral (**d**) retinas. (GCL: ganglion cell layer; IPL: inner plexiform layer; INL: inner nuclear layer; OPL: outer plexiform layer; ONL: outer nuclear layer; IS: inner segment; OS: outer segment; RPE: retinal pigment epithelium; CC, choriocapillaris).

**Figure 2:**
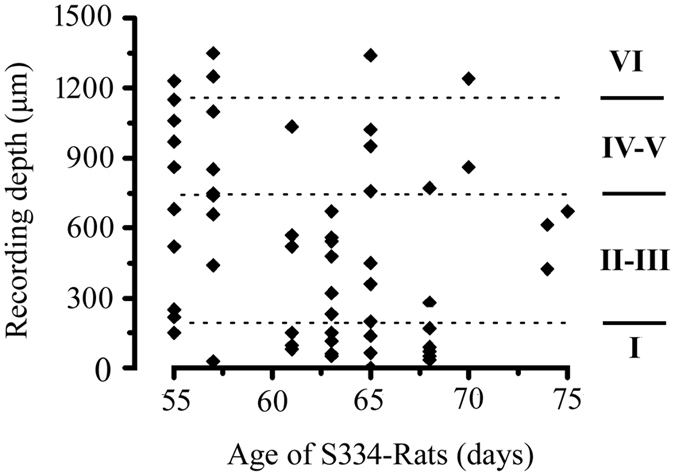
Depth and age distribution of the S334ter-3 rats. Four layers can be roughly confined according to the depth below the cortical surface.

In a previous study^10^, a progressive decrease in the thickness of the outer nuclear layer (ONL) was observed in S334ter-3 rats. Figure 1 shows light microscopy with hematoxylin and eosin (H&E) staining from both S334ter-3 and LE retinas (P65). The retina thickness from the S334ter-3 rats was less than that of the age matched control group (a: LE centre retina; b: LE peripheral retina; c: S334ter-3 centre retina; d: S334ter-3 peripheral retina). Specifically, the outer plexiform layer (OPL), outer nuclear layer (ONL) and the inner and outer segments (IS/OS) of the photoreceptor almost disappeared. We found that these layers were notably atrophied in the S334ter-3 retinas at P65 compared to the LE retinas. Upon reconstructing the recording tracks, a good correspondence was obtained from the distance between the brain surface and the recording sites using the micromanipulator. Figure 2 shows the laminar and age for all S334ter-3 neurons in the experiments. Four layers can be roughly visualized based on the depth below the cortical surface. The most superficial units (layer I) could be successfully isolated from a depth of 200 μm below the cortical surface. Layers II & III were located at a depth of 200–750 μm and Layer IV & V was located at a depth of 750–1150 μm. The last layer (layer VI) ranges in depth from 1150–1500 μm.

### Orientation selectivity alterations in the degenerated group

**Figure 3:**
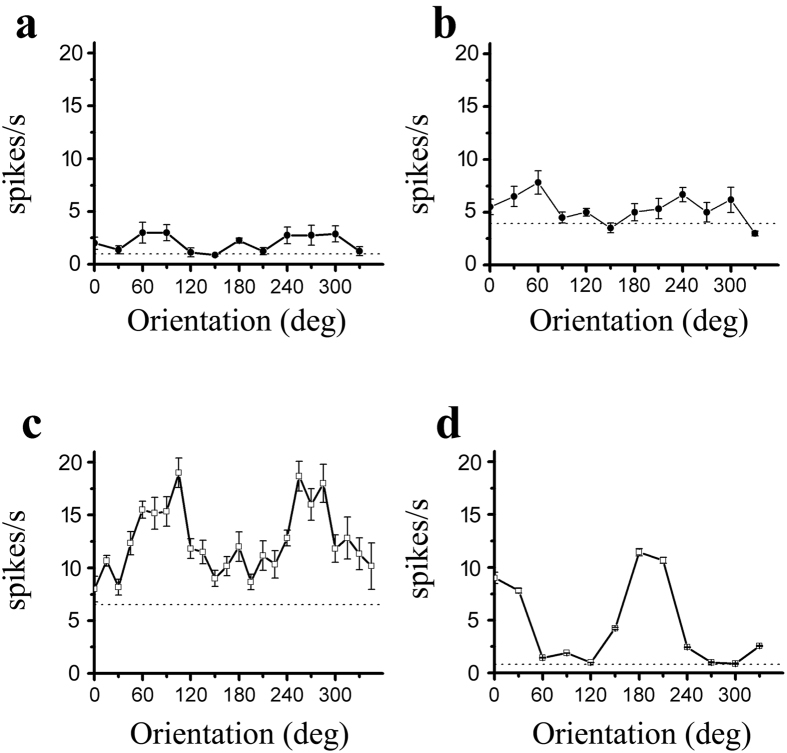
Examples of orientation tuning curves from S334ter-3 rats (**a**) P65, (**b**) (P61) and LE rats (**c**,**d**). The dotted line indicates the spontaneous response. Error bars represent the SEM values.

**Figure 4:**
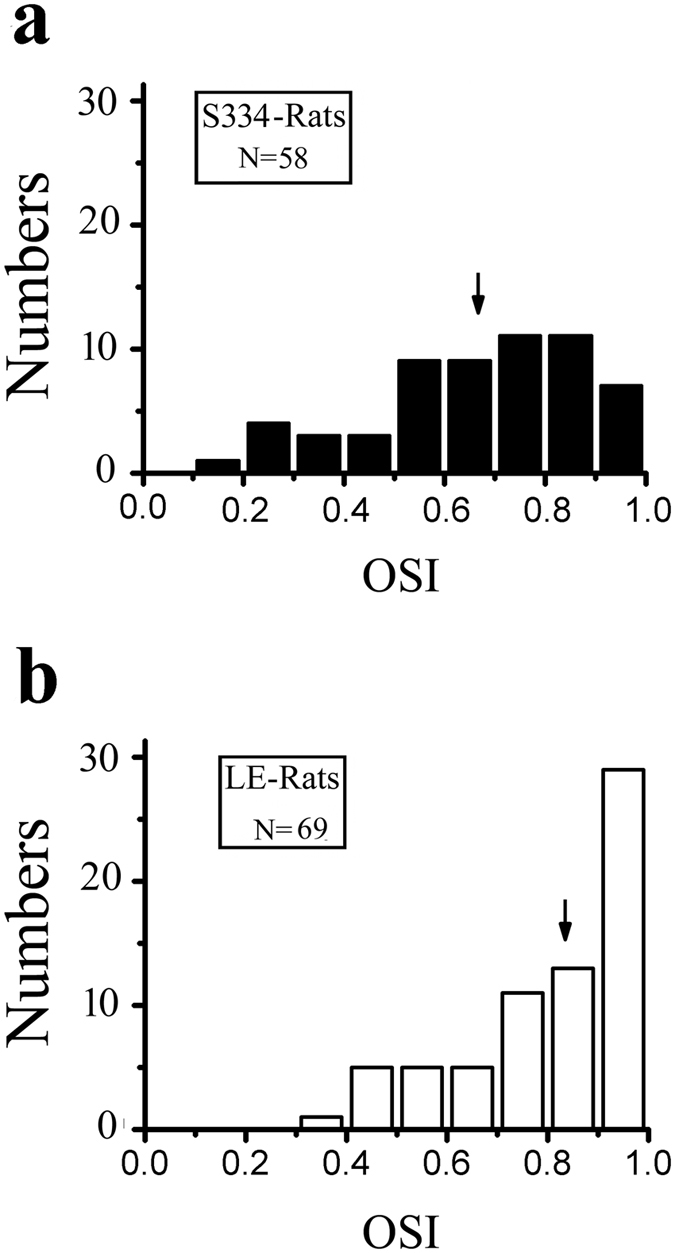
The OSI distributions from S334ter-3 rats (**a**) and LE rats (**b**). The solid arrow indicates the average OSI for each population (**a**) 0.67; (**b**) 0.82.

**Figure 5:**
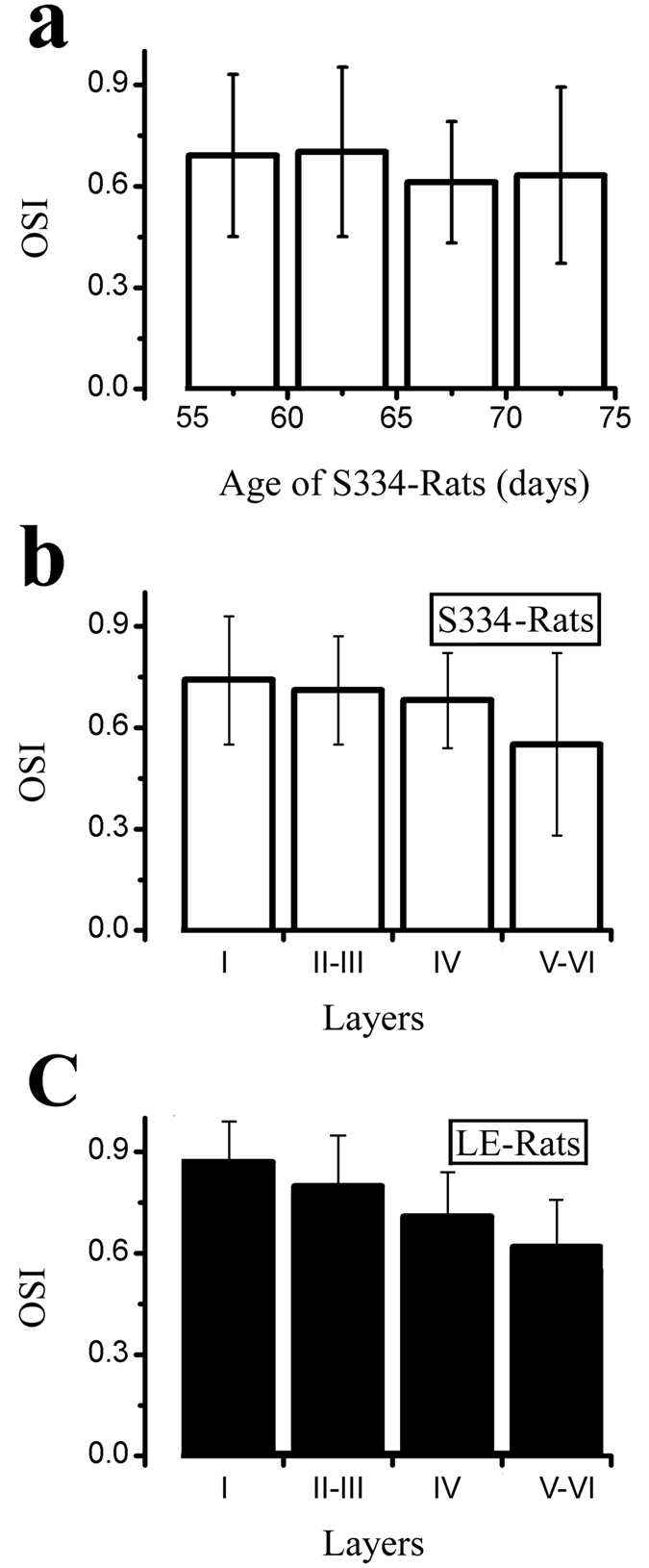
The OSI distribution from different ages and laminar layers of the S334ter-3 rats. (**a**) Histogram of OSIs at different ages in S334ter-3 rats. (**b**,**c**) Histogram of OSIs at different laminar layers in S334ter-3 rats (**b**) and LE rats (**c**).

We measured orientation tuning using sinusoidal gratings with different orientations in which drifting sinusoidal gratings were presented with a fixed spatial frequency of 0.04 cpd, a temporal frequency of 2 Hz, and a contrast of 100%. Figure 3 shows the orientation tuning from the S334ter-3 rats (a, b) and LE rats (c, d). To quantify the orientation tuning, we fit the firing rate as a function of the orientation by the sum of two Gaussians. Because only a few cells in the S334ter-3 rats had a good orientation tuning curve, the fitting error was larger than 0.5. Thus, we used an alternate method to quantify the orientation selectivity. The orientation selectivity index was (OSI) = (R_opt_ − R_ver_)/(R_opt_ − R_spon_), where R_opt_ and R_ver_ were responses to optimal and orthogonal to optimal orientation, respectively, and R_spon_ was the spontaneous activity. The OSI indicated the variation in the response between the orthogonal to the optimal orientation. The larger the OSI, the better the orientation selectivity. Figure 4 shows the OSI distribution for the S334ter-3 (a) and LE rats (b). The average OSI was 0.67 ± 0.23 for the S334ter-3 rats (n = 58) and 0.82 ± 0.19 for the LE rats (n = 69). There was a significant difference in the OSI between the degenerated group and the control group (P = 0.0004, Mann-Whitney U test). Figure 5a shows the relationship between the OSI and the age of the S334ter-3 rats; the average OSIs were 0.69 ± 0.24 (P55–59), 0.7 ± 0.25 (P60–64), 0.61 ± 0.18 (P65–69), and 0.63 ± 0.26 (P70–75) (P > 0.05 for all comparisons, Mann-Whitney U test). Figure 5b,c show the OSI distributions for each layer in the S334ter and LE rats. The mean OSI gradually decreased in the deeper laminar layers in both the S334ter and LE rats. Significant differences were confirmed between the OSIs of neurons recorded at layers V-VI and the other layers (I & V-VI: P < 0.01; II-III & V-VI: P < 0.05; IV & V-VI: P < 0.05, Mann-Whitney U test).

### Alterations in spatial and temporary tunings in the degenerated group

**Figure 6:**
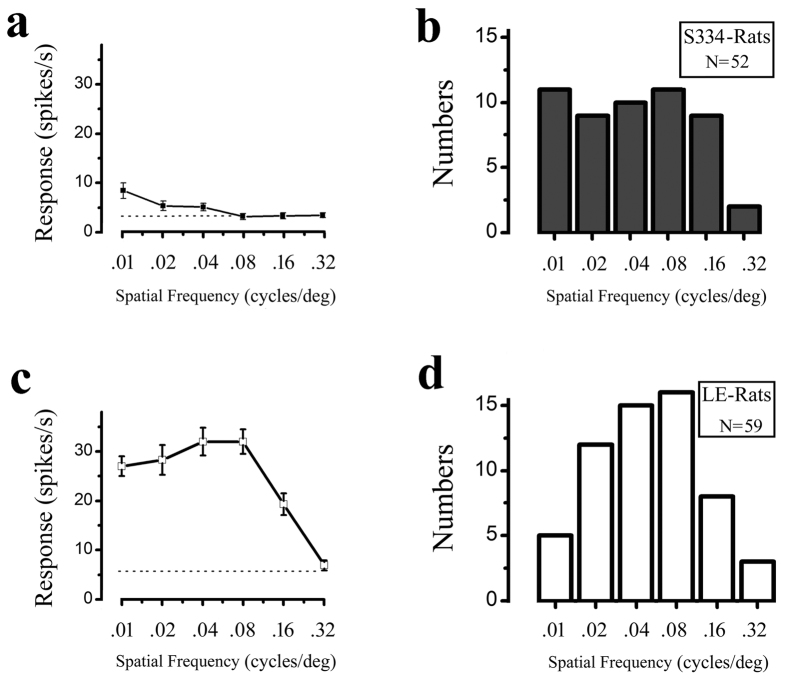
Distribution of the optimal spatial frequency for the S334ter-3 and LE rats. Example ((**a**) P61) and histogram (**b**) of the optimal spatial frequency from S334ter-3 rats. Example (**c**) and histogram (**d**) of the optimal spatial frequency from LE rats. The dotted line indicates the spontaneous response. Error bars represent the SEM values.

The spatial tuning for each cell was examined using gratings in which the SF was varied while the other parameters were kept optimal. We used six different spatial frequencies to measure the cell’s responses. Examples of the cell’s spatial frequency tuning from the S334ter-3 and LE rats are shown in Fig. 6a,c, respectively. Figure 6b,d show the distribution of the optimal spatial frequencies for the S334ter-3 rats and LE rats, respectively. We classified the optimal spatial frequencies as the low spatial frequency (≤0.04 cpd) and high spatial frequency (≥0.08 cpd). Twenty cells (38.5%) from the S334ter-3 rats and 17 (28.8%) from the LE rats had an optimal response at the low frequency (≤0.04 cpd). The other cells had an optimal spatial response at the high spatial frequency (≥0.08 cpd). Although more S334ter-3 neurons had optimal responses at the low spatial frequency, there was no significant difference in the distribution of the optimal spatial frequencies in the high and low spatial frequencies in the degenerated group and the control group (P = 0.282, Chi-square test).

**Figure 7:**
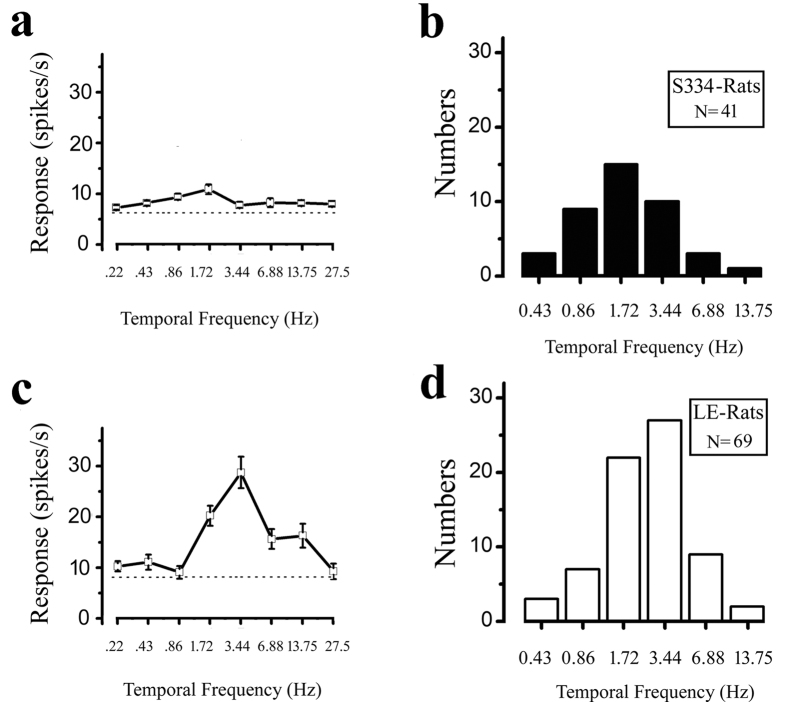
Distribution of the optimal temporal frequency for the S334ter-3 and LE rats. Example ((**a**) P65) and histogram (**b**) of the optimal spatial frequency from S334ter-3 rats. Example (**c**) and histogram (**d**) of the optimal spatial frequency from LE rats. The dotted line indicates the spontaneous response. Error bars represent the SEM values.

Temporary frequency tuning was examined using gratings in which the temporary frequency varied while the other parameters were kept optimal. We used eight different temporary frequencies to measure the responses of rat V1 neurons. Examples of the cell’s temporal frequency tuning are shown in Fig. 7a,c. Figure 7b,d show the optimal temporary frequency distributions. We classified the optimal temporary frequency as the low temporary frequency with a frequency less than 1.72 Hz and as the high frequency with a frequency higher than 3.44 Hz. The results showed that 27 cells (65.9%) in the S334ter-3 rats and 32 (46.4%) cells in the LE rats had optimal responses at ≤1.72 Hz. The other cells had optimal responses at the high temporary frequency. More cells in the S334ter-3 rats had their optimal temporary frequencies at the low temporary frequency compared to the control group. There was a significant difference in the optimal temporary frequencies distributed in the high and low temporary frequencies between the S334ter-3 and LE rats (P = 0.048, Chi-square test).

### Decrease in the receptive field size in the degenerated group

**Figure 8:**
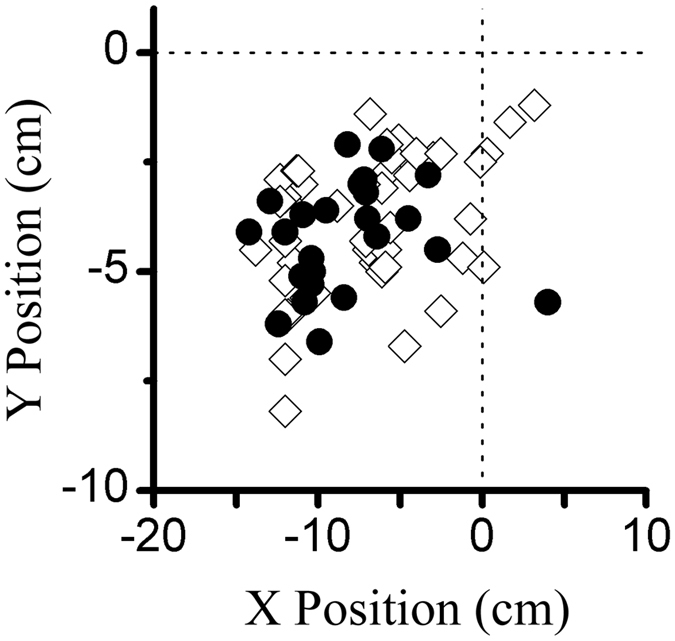
The distribution of the receptive field centre. The solid circles and hollow diamonds represent cells in the S334ter-3 and LE rats, respectively. The dotted lines indicate the centre of the screen display.

**Figure 9:**
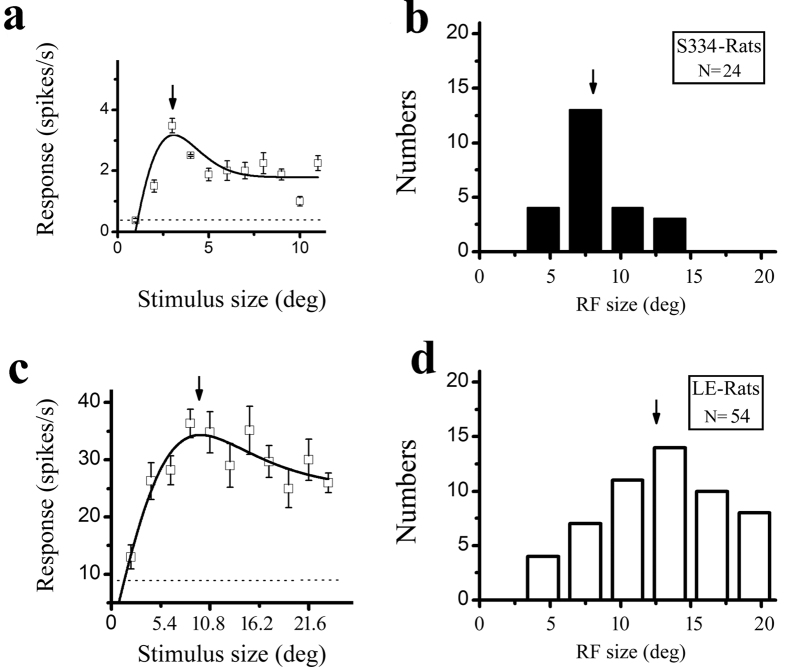
Distribution of the receptive field for the S334ter-3 and LE rats. Example ((**a**) P68) and histogram (**b**) of the receptive field from S334ter-3 rats. Example (**c**) and histogram (**d**) of the receptive field from LE rats. The arrow in (**a**,**c**) indicates the size of the receptive field. The arrow in (**b**,**d**) indicates the average of the population (**b**) 8.1; (**d**) (12.9). The dotted line indicates the spontaneous response. The error bars represent the SEM values.

The RFs are partially dependent on the eccentricity of their locations in the visual field and partially dependent on the balance between the excitatory and inhibitory regions. In the present experiment, we could not map the eye vascular figure for each eye to confirm the location of the central fovea. We located the centre of the classical receptive field (CRF) by placing a narrow sine-wave grating patch at successive positions in a random sequence along the axes perpendicular or parallel to the optimal orientation of the cell and then measuring the response to its drift. The centre of the CRF was defined as the peak of the response profiles for both axes. Figure 8 shows the distribution of the receptive field centre for the S334ter-3 (solid circles) and LE rats (hollow diamonds). Their centres were located in an overlapping area. Then, we measured the spatial extent over which the responses summated by centering a grating stimulus over the centre of the CRF and increasing the diameter of the stimulus patch. We measured how the response amplitude depended on the stimulus diameter. An example of a cell’s spatial summation tuning from a S334ter-3 rat and a LE rat is shown in Fig. 9a,c, respectively. The response rose to a peak and then decreased as the stimulus diameter increased. The solid line through the data points was the best-fitting difference of the integrals of the Gaussian function. From this function, we extracted two parameters: the peak response diameter (stimulus diameter at which the response was maximal) and a suppression index (1-asympotic response/peak response). The distributions of the peak response function are shown in Fig. 9b (S334ter-3 rats, n = 24) and Fig. 9d (LE rats, n = 54). The average RFs were 8.1 ± 2.1 degrees for the cells in the S334ter-3 rats and 12.9 ± 4.4 degrees for the cells in the LE rats. There was a significant difference in the RFs between the S334ter-3 and LE rats (P < 0.001, Mann-Whitney U test). No significant difference was found in the RFs between the different layers (P > 0.05 for all comparisons, Mann-Whitney U test).

Most rat V1 neurons had significant surround suppression. We used a suppressive index (SI) to quantify the strength of the surround suppression. An index of 0 indicated a cell whose responses grew to an asymptote as the stimulus size increased, whereas an index of 1 indicated a cell whose responses could be completely abolished by large stimuli. The average SI was 0.41 ± 0.22 (n = 24) for the cells in the S334ter-3 rats and 0.37 ± 0.32 (n = 54) for the cells in the LE rats. We found no significant difference in the SI between the S334ter-3 and LE rats (P > 0.05, Mann-Whitney U test).

## Discussion

The present study focused on the comparison between receptive field properties in V1 cells from S334ter-3 and LE rats. The results showed that the V1 cells in the S334ter-3 rats exhibited weaker orientation selectivity, lower optimal spatial and temporal frequency values, and smaller RFs compared to the cells in LE rats.

### Weakening of orientation tuning and visual sensitivities during retinal degeneration

Previous studies reported that most neurons in the LE rats were categorized as orientation selective and that the majority of orientation-selective cells had orientation tuning^22^,^23^,^24^. Our finding in the control group was consistent with these previous studies. We found that orientation selectivity was significantly decreased in the degenerated group. Thus, we investigated whether the change in OSI might be caused by reduced visual sensitivities during RD. Sekirnjak *et al.* reported the direct effects of photoreceptor degeneration on RGCs in the P23H-1 rat. The authors found the RFs were diminished and their strength decreased relative to the noise^16^. Recordings in the superior colliculus of RCS rats also showed that visual sensitivities in response to light flashes were progressively reduced with age^13^,^25^. Pu *et al.* reported detailed RF properties in the degenerating retina and found that the SNR and RF sizes decreased with advancing degeneration^26^.

Hubel and Wiesel first described orientation selectivity in the neurons of cat V1 and proposed an elegant and direct model. The model represented the feedforward model in its simplest form and explained orientation selectivity based solely on the organization of the feedforward input to a simple cell^27^. The reduced photoreceptors and visual sensitivities to light weakened the feedforward inputs to the V1, which would lead to a significant decrease in the orientation tuning selectivity. An investigation in LE rats reported that the spatial frequencies of V1 neurons covered a range from 0.01–1.2 cpd with an optimal response at 0.08 cpd and a temporal frequency with a range from 0.43–13.75 Hz with an optimal response at 3 Hz^19^. During degeneration, our results showed that cells in the S334ter-3 rats tended to respond better at a lower spatial and temporal frequency compared to the control group. This result is most likely due to the reduction in the spatial and temporal resolution caused by damage in the photoreceptors.

### Shrinking and weakening of the receptive field during retinal degeneration

Substantial evidence has indicated that geniculocortical feedforward afferents to V1 primarily integrate signals within the classical receptive field. In the present study, we discovered that the RFs in S334ter rats were smaller than those in LE rats. Therefore, we investigated the decrease in RFs caused by the large loss of photoreceptors. The retina contains a complex network of cells that can be divided into an estimated 60–80 cell types, including 3–4 photoreceptors and 15–20 ganglion cells^28^,^29^,^30^. Most cells have conventional centre-surround receptive fields. The simple model for retinal ganglion cells includes a linear filter (receptive field) with a centre-surround organization and a half-wave rectifying nonlinearity^31^. Images that resemble the filter produce large firing rate responses, whereas images that resemble the inverse of the filter or have no similarity with the filter produce no response. Thus, we inferred that the shrinkage in RFs in V1 occurred due to rectifying nonlinearity. Only a few photoreceptors remaining in the retinal ONL may produce lower output responses from the linear filter. Rectifying nonlinearity may lead to a significant decrease in the response of the output to the LGN. We inferred that this decrease could cause the smaller RFs in V1 and the low peak firing rate in the spatial summation test.

Previous studies in many species (monkeys, cats, and rats) have demonstrated that the classical receptive field exhibits a 2- to 3-fold increase as the contrast drops from high to low^32^,^33^. The contrast-dependent variations in RFs primarily derive from intracortical connections within V1 or feedback projections from the extrastriate cortex^34^,^35^. At high contrast, shrinkage of the CRF may result in an improvement in the spatial resolution of visual detection and the capacity to precisely localize features of an image. At low contrast, the expansion of spatial summation produces increased sensitivity and a better detection capability for weak signals by sacrificing spatial resolution. In the present study, we used high stimulus contrast to compare the RFs in S334ter-3 and LE rats and found smaller RFs in the S334ter-3 rats. A V1 neuron summated the input between the excitation and inhibition balance. Because smaller RFs were detected in the S334ter-3 rats, the reduction in the inhibition was much less than the reduction in the excitation. We inferred that the new balance between excitation and inhibition might improve visual cognition in RD.

### Surround suppression during retinal degeneration

More recent studies revealed that many V1 neurons in rats exhibited an increased stimulus diameter and decreased response^36^,^37^,^38^,^39^. In the study of Girman *et al.*^19^, 54 out of 158 cells exhibited clear inhibition when presented with stimuli extending beyond their RF centre. In this study, we also used the spatial summation test to measure the RF and SI, although only a few neurons had strong surround suppression. Our results showed that the mean SI was 0.41 ± 0.22 for cells in the S334ter-3 rats and 0.37 ± 0.32 for cells in the LE rats (P > 0.05). Previous investigations of surround suppression showed that the mean SI of V1 cells was 0.44 in cats^40^ and 0.58 in monkeys^41^, both of which were larger than the response in rat neurons. Substantial evidence indicated that intracortical connections within the V1 or feedback projections from the extrastriate cortex might play crucial roles in the origin of surround suppression. Our results indicated a similar SI in the visual cortices of S334ter-3 and LE rats under RD, which also maintained normal functions and some degree of plasticity.

## Methods

### Animal Preparation and Maintenance

All animals were obtained from the Laboratory Animal Services Centre of Chinese University of Hong Kong and housed in animal facilities at the City University of Hong Kong. All experimental procedures were approved by the Animal Subjects Ethics Sub-Committees of City University of Hong Kong and the Health Department of Hong Kong Special Administrative Region. All methods were carried out in accordance with the approved welfare guideline of the Animal Subjects Ethics Sub-Committees of City University of Hong Kong and the Health Department of Hong Kong Special Administrative Region. Acute experimental recordings were made from 11 S334ter-line-3 (S334ter-3) and 10 Long-Evans (LE) immature rats that were 55–85 days of age. S334ter-line-3 rat litters were obtained by breeding transgenic homozygotes (with two copies of the mutant transgene) with Long-Evans rats. Hence, the experimental rats (S334ter-3) carried one copy of the mutant transgene. LE and S334ter-3 rats (2–3 months of age) were used as the WT and retinal degeneration model, respectively. The surgical procedures were similar to those described in the previous work^22^. The animal was anesthetized with an intraperitoneal injection of Ketamine-Xylazine combination (Ketamine: 70 mg/kg and Xylazine: 7 mg/kg; Alfasan International B.V., Holland) initially; then, 2% isoflurane (RWD Life Science, Shenzhen, China) was applied during the recording. After the craniotomy, a bone screw was fixed in the skull and a tungsten electrode was placed on the surface of the primary visual cortex as the ground and recording electrodes, respectively. The exposed cortical surface was covered with 2% agar to prevent drying and reduce the brain pulsation caused by breathing; then, a shield was used to attenuate environmental interference. The animal was maintained at 38 °C using a warm pad, and ocular solution was regularly applied to keep the eyes moist throughout the procedures, including the recording session.

### Recording and visual stimulation

The A-M Systems 3600 (A-M Systems, US) and CED Micro 1401-3 (Cambridge Electronic Design, UK) were used as the amplifier and data acquisition system, respectively. Tungsten-in-glass microelectrodes were used to record single units extracellularly. As the microelectrode was advanced slowly by the oil hydraulic micromanipulator (MO-10, Narishige, Japan), various stimuli were displayed across the animal’s visual field to activate the cells. Single-unit activity was filtered from 0.5–5 kHz and then sampled at 40 kHz using an acquisition card (National Instrument, USA). Stimuli were presented on a Dell monitor (screen size 35 × 26 cm, frame rate 60 Hz, and resolution 800 × 600 pixels) positioned 23 cm from the rat’s eye (the screen occupied 75 × 58° in the visual field). The contrast of the grating was 100%, and the mean luminance was 26 cd/m^2^. Preliminary tests showed that spatial resolution was not improved by varying the distance or by placing corrective lenses in front of the eye to correct for the previously reported hypermetropia^42^,^43^ or myopia^44^,^45^ of the rat eye. All measurements were made during the stimulation of the rat’s dominant eye. Under computer control, the grating orientation and the spatial and temporal frequencies were matched to the preferred parameters of the cell under study and real-time analyses of the responses were performed. Then, we located the centre of the CRF by placing an arrow sine-wave grating patch at successive positions in a random sequence along the axes perpendicular or parallel to the optimal orientation of the cell and then measuring the response to its drift. The centre of the CRF was defined as the peak of the response profiles for both axes. Once the receptive field centre was established, we performed size-tuning measurements. Circular drifting sinusoidal grating patches of different diameters were used as the stimuli. The empirical size of the CRF was defined as the stimulus radius at which the size-tuning curve reached its peak value (for the cells with surround suppression) or 95% of the peak value (for cells without surround suppression).

Finally, we measured the cells’ responses to different stimulus contrasts in the receptive field size. Each experiment consisted of several blocks of trials in which stimuli with particular parameter values were presented in a random order to minimize the possible effects of response variability. The results of several repeated blocks (typically 5 but not less than 3) were averaged. Responses to a uniform field of averaged luminance were also recorded to measure the neuron’s spontaneous firing rate. Peri-stimulus time histograms (PSTHs) of unit responses were generated and analysed on-line using custom-made software. Then, the rat was euthanized with an overdose of dorminal (300 mg/kg).

### Histology

After the recording, the eyeballs of both the S334ter-3 and LE rats were enucleated and fixed in 10% formalin in 0.1 M phosphate buffer (pH 7.4) at 4 °C overnight. The samples were dehydrated with a graded series of ethanol and xylene and subsequently embedded in paraffin wax. Retinal sections (5 μm) with pupil-optic nerve position were stained with H&E. The retinal sections were imaged with a light microscope to assess the status of the different retinal layers.

### Data Analysis

For each neuron, we calculated an orientation selectivity index^19^ (OSI) to quantify the orientation tuning and direction selectivity of each neuron:


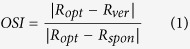


in which *R*_opt_ is the response to optimal orientation, *R*_ver_ is the response to orthogonal to optimal orientation, and *R*_spon_ is the spontaneous firing level (discharge rate with a uniform field of average luminance).

The spatial summation curves for all recorded cells were fitted using a difference of Gaussians model. The two Gaussians are considered to be concentrically overlapping, and the summation profile can be represented as the difference of the two Gaussian integrals^32^:


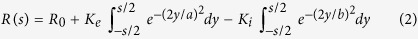


where *R*_*0*_ is the spontaneous firing rate and each integral represents the relative contribution from putative excitatory and inhibitory components. The excitatory Gaussian is described by gain (*K*_*e*_) and a space constant *a* and the inhibitory Gaussian by its gain (*K*_*i*_) and a space constant *b*.

The suppression index (SI) measurement was estimated from the fitted curve and defined as follows:


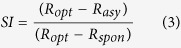


where *R*_*opt*_ is the maximum response and *R*_*asy*_ is the asymptotic response. *R*_*spon*_ is the spontaneous firing level when no visual stimuli is given. When SI = 0, there is no suppression and the response is either increasing or reaching a plateau. When SI = 1, the response is suppressed to the spontaneous firing level. The SI of most cells was between 0 and 1.

All values were optimized to provide the least mean square error for the data. All fitting procedures were conducted with the MATLAB optimization toolbox using the CONSTR and FMINCON nonlinear least-squares functions. To evaluate how well our experimental data fit the model, the goodness of each fit was established by calculating the mean fraction error defined as follows:


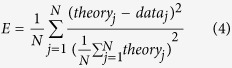


where *theory*_*j*_ and *data*_*j*_ are the expected response theory and the experimental response data to the *j*th stimulus size, respectively. The error in fit found in the present study ranged from 0.004 to 0.15 with a mean error of 0.036 across the population.

All population values given below are expressed as the means plus or minus the standard error of the mean.

## Additional Information

**How to cite this article**: Chen, K. *et al.* Electrophysiology Alterations in Primary Visual Cortex Neurons of Retinal Degeneration (S334ter-line-3) Rats. *Sci. Rep.* **6**, 26793; doi: 10.1038/srep26793 (2016).
